# CONSTRUCTION AND VALIDATION OF THE NEONATAL NUTRITIONAL RISK SCREENING TOOL

**DOI:** 10.1590/1984-0462/2021/39/2020026

**Published:** 2020-12-18

**Authors:** Rayssa Caroline de Almeida Silva Silvino, Vanessa Camargo Trida, Amparito Del Rocío Vintimilla Castro, Lenycia de Cassya Lopes Neri

**Affiliations:** a Universidade de São Paulo, São Paulo, SP, Brazil.

**Keywords:** Newborn, Neonatal screening, Infant nutrition, Recém-nascido, Triagem neonatal, Nutrição do lactente

## Abstract

**Objective:**

To develop and validate both the content and reliability of the Neonatal Nutritional Risk Screening Tool (FARNNeo).

**Methods:**

Methodological study, convergent care. The instrument was built prior to the literature review and was analyzed by eight judges, during three cycles of the Delphi technique. The judges assessed their relevance and clarity with responses on the Likert scale with three levels, in addition to suggestions. The validation of the instrument was calculated using the agreement rate and content validity index (CVI). After content validation, the instrument was applied by four assisting nutritionists to verify reliability, using Cronbach`s alpha coefficient and the agreement between the evaluators by the *Kappa* coefficient.

**Results:**

All items of the instrument`s content reached the minimum agreement rate (90%) and/or CVI (0.9), except for item three, which in the first cycle obtained CVI 0.77 and 40% of agreement and, in the second cycle, CVI 0.75 and 38% agreement. At the end of the third cycle, all items had CVI values above 0.9. In the instrument application, alpha of 0.96 and *Kappa* of 0.74 were obtained, which reflect adequate values of internal consistency and agreement between the evaluators.

**Conclusions:**

FARNNeo proved to be reliable, clear, relevant, and reproducible for tracking early nutritional risk, systematizing the care of Brazilian newborns admitted to an intensive care unit.

## RESUMO

**Objetivo:**

Desenvolver e validar o conteúdo e a confiabilidade da Ferramenta de Avaliação do Risco Nutricional Neonatal (FARNNeo).

**Métodos:**

Estudo metodológico, convergente assistencial. O instrumento foi construído previamente à revisão da literatura e analisado por oito juízes, durante três ciclos da técnica Delphi. Os juízes avaliaram sua pertinência e clareza com respostas na escala Likert com três níveis, além de sugestões. A validação do instrumento foi calculada pela taxa de concordância e índice de validade de conteúdo (IVC). Após a validação do conteúdo, o instrumento foi aplicado por quatro nutricionistas assistenciais para verificar a fidedignidade, utilizando o coeficiente de alfa de Cronbach e a concordância entre os avaliadores pelo coeficiente *Kappa*.

**Resultados:**

Todos os itens do conteúdo do instrumento alcançaram o valor mínimo da taxa de concordância (90%) e/ou IVC (0,9), com exceção do item três, que no primeiro ciclo obteve IVC 0,77 e 40% de concordância e, no segundo ciclo, IVC 0,75 e 38% de concordância. No fim do terceiro ciclo, todos os itens obtiveram valores de IVC acima de 0,9. Na aplicação da ferramenta, obteve-se alfa de 0,96 e *Kappa* de 0,74, que refletem valores adequados de consistência interna e concordância entre os avaliadores.

**Conclusões:**

A FARNNeo mostrou-se confiável, clara, pertinente e reprodutível para rastreamento do risco nutricional precoce, sistematizando o atendimento de recém-nascidos brasileiros internados em centro de terapia intensiva.

## INTRODUCTION

During the first days of life, newborns (NB) experience an intense period of adaptations. During their intrauterine life, consequences may be reflected on their conditions of birth if they are exposed to challenging situations. Conditions vary from weight, gestational age, speed of postnatal growth, and evolutive results.^1^ ^,^ ^2^ ^,^ ^3^ An inadequate nutritional status poses negative implications for these children and determines consequences for their health and development. Malnutrition contributes to the increase in morbidity and mortality, time of hospitalization, hospital costs, and a worse quality of life of children and their families. ^4^

Even with the association of hospital malnutrition and risks of adverse events, this remains an underestimated problem, which is often unknown. Seen that, early detection of nutritional depletion during hospitalization is essential, creating a need for evaluating and strictly following up the patient’s nutritional status to prevent malnutrition and its consequences. For that purpose, the early detection of malnutrition and risk assessment must be conducted, making the most intensive intervention feasible for the multiprofessional team to preserve and recover patients, preventing possible complications.^5^

Nutritional screening of NB can identify the classification of nutritional risk, and is essential to follow up and verify the degree of development and the probability of morbidity and mortality associated to their nutritional status. Applying such screening is essential at the time of hospital admission because it detects the need for an early intervention and better directs the actions to be taken. Nutritional follow-up is an important ally in children’s growth and development, mainly when it comes to preterm newborns with associated disease, which presents specific nutritional needs that, if not identified, can cause problems to a healthy development.^6^ ^,^ ^7^ ^,^ ^8^

The tools of nutritional screening identify deviations and risks of complications related to nutritional status, and its applications may help anticipate nutritional guidelines to prevent sequels, which makes it a relevant strategy to identify the need for a more detailed care and a proper institution for nutritional support, besides offering a better destination to and organization of resources.^9^ ^,^ ^10^

In this context, tools with that purpose were identified, but they were limited and inadequate for Brazilian newborn patients admitted to the Neonatal Intensive Care Center (NICC) of a tertiary hospital. This fact may be due to the methodology applied, lack of validation, its non-reproducibility, the use of subjective criteria, lack of good sensitivity, its specificity, or its non-practical application. Therefore, the objective of the present study was to create and validate the content and reliability of the Neonatal Nutritional Risk Screening Tool (*Ferramenta de Avaliação do Risco Nutricional Neonatal* - FARNNeo).

## METHOD

This is a methodological, convergent-care study of a quantitative nature, developed in two stages: construction and validation of both content and reliability of a neonatal nutritional risk screening tool, which will be called FARNNeo, to be applied by the multidisciplinary team working at NICC. In methodological research, the goal is to develop reliable and accurate tools that can be applied by the multiprofessional team. ^11^ ^,^ ^12^

This study was carried out at the Neonatal Intensive Care Center 2 (NICC2) of Instituto da Criança, Hospital das Clínicas, Medical School of USP (ICR-HCFMUSP), from May to November 2019. It was approved by the Research Ethics Committee (*Comitê de Ética em Pesquisa* - CEP) of the Faculty of Medicine of Universidade de São Paulo, with Certificate of Presentation for Ethical Appreciation (*Certificado de Apresentação para Apreciação Ética* - CAAE) No. 10127219.5.0000.0068 and opinion No. 3.312.967.

The first step was to build the tool’s content. A bibliographic survey was carried out to identify the main parameters that impact the nutritional risk of a newborn.^13^ ^,^ ^14^

The tool was built with four questions and, for each item of the questions, a score was attributed, according to its impact on nutritional status. The items that express greater severity have a higher score. At the end, from the sum of the scores, screening classifies NB of low (0 point), medium (1 to 3 points), or high (≥4 points) nutritional risk. The questions assess the following aspects:

1. Gestational age of birth, which classifies newborns into: Extreme preterm infants, preterm infants, and term infants.
2. Evaluation of birth weight, which classifies newborns as: extremely low weight, very low weight, low weight, and adequate weight.
3. Disease and/or clinical condition with high nutritional risk.
4. Nutritional support to which the newborn is submitted at the time of screening: fasting without nutritional therapy, exclusive parenteral nutrition, exclusive or mixed enteral nutrition, and exclusive oral route.

For tool validation, the Delphi technique was used, with three cycles. The method is a strategy widely used to obtain expert evaluation on a given subject, without direct communication.^15^ The tool was analyzed by a group of experts who acted as judges, composed of ten professionals with the following inclusion criteria: being a doctor, nutritionist, or nurse; having the title of specialist, master or doctor; working with the theme, and working in the areas of assistance, management or teaching and research, with more than ten years of training and professional experience. Professionals were invited to participate via e-mail. We sent a form prepared on Google Forms, which initially contained a brief text explaining research, and an informed consent form for participation. After that, we presented the built screening tool and the description of the tool’s items.

Each item was assessed for relevance and ease of understanding. Responses were presented on a Likert scale, with scores from one to three, in which 1=no, 2=maybe, and 3=yes. By the end of each question, we provided a field for suggestions so that the judges could give feedback on the questions, as well as enrich the content with timely aspects.^16^

For content validation, agreement rate and content validity index (CVI) were calculated. The latter is widely used to calculate the consensus among judges, because it measures the agreement on the aspects evaluated.^15^ Agreement rate was calculated by dividing the number of positive responses by the total number of responses. CVI can be calculated by following four steps. In the first, based on scores given by judges (1 to 3), the average of scores for each item is calculated. In the second, based on the average, the initial CVI for each item is calculated, dividing it by the maximum value that the question could receive for relevance or clarity. In the third, the error of each item is calculated to discount possible biases by the judges. To obtain the error, one (1) is divided by the number of judges, raised by the same number of evaluators. In the fourth, the final CVI of each item can be determined based on the subtraction of the initial CVI by the error. The acceptable consensus rate in this study was 90%, or CVI of 0.9, for each item analyzed to be considered valid.

**Figure 1:**
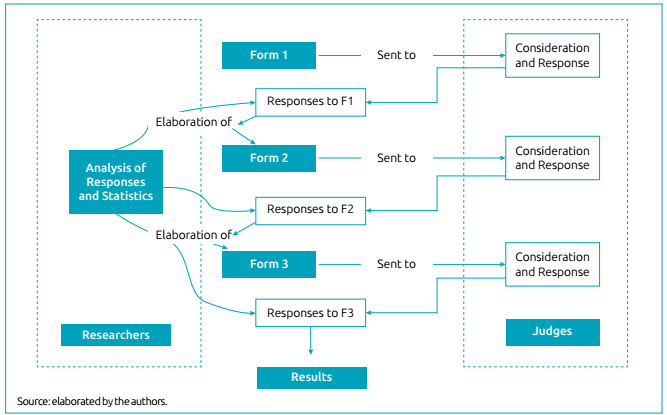
Illustration of Delphi cycles.

The validation of the tool’s content went through three cycles with specialists, so that the minimum CVI value could be reached for all items. Responses obtained were organized in an Excel spreadsheet, with numerical and subjective information filled in by the judges. In the flowchart, illustrated in Figure 1, all Delphi phases used in this study are observed.

In the first cycle, the ten judges responded and made suggestions. The tool was reformulated according to opinions, after discussion among researchers. The tool for the analysis of judges was sent again. In the second cycle, eight of them responded; the other two were disregarded for the next cycle. In the third cycle, the eight judges responded. Calculations were carried out and the consensus initially proposed was obtained.

After validating the tool’s content, four nutritionists who work in the care area and who had not participated in the previous stages of the study were invited to apply the tool to 16 newborns admitted to the NICC2 of ICR-HCFMUSP, individually and without contact between them during application. A table was made available with data on gestational age at birth, birth weight, disease and/or clinical condition, and nutritional therapy at the time of screening. With the tool validated, the classification of patients as low, medium, or high nutritional risk was requested.

In this stage, reliability was verified with internal consistency, using Cronbach’s *alpha* to classify it as: almost perfect, when it reached values above 0.8; substantial, for values from 0.61 to 0.8; moderate, from 0.41 to 0.6; reasonable, between 0.21 and 0.4; and of small consistency, when less than 0.21.^17^ The test was performed using the MedCalc program, version 17.8.6, in a Windows XP environment.

To assess agreement between the evaluators (reproducibility), Kappa Fleiss coefficient was used, characterized by different ranges, in which: values above 0.75 represent excellent agreement; values under 0.40 represent low agreement; and values between 0.40 and 0.75 indicate satisfactory agreement.^18^ The test was performed using the Stata program, version 14.0.

## RESULTS

Table 1 shows the characterization of the judges who participated in the three Delphi cycles, according to sex, place of professional practice, title of professional qualification, field of knowledge, and area of professional performance.

**Table 1:** Characteristics of the judges who participated in the validation of the tool’s content. São Paulo City, São Paulo State, Brazil, 2020.

	No.	%
Gender		
F	5	62.5
M	3	37.5
Total	8	100.0
Place of work		
São Paulo City	8	100.0
Total	8	100.0
Degree of professional qualification		
Post doctoral	1	12.5
Doctoral	3	37.5
Masters	1	12.5
Specialization	3	37.5
Total	8	100.0
Field of knowledge		
Physician	3	37.5
Nurse	2	25.0
Nutritionist	3	37.5
Total	8	100.0
Professional area		
Clinical care	3	37.5
Teaching and research	2	25.0
Administration	3	37.5
Total	8	100.0

Table 2 shows the results of the three cycles as to the validation of the tool’s content. All items were considered relevant by the judges since the first Delphi cycle. Regarding clarity, item three did not reach an initial consensus.

**Table 2:** Agreement rate of judges and content validity index (CVI) in the tool’s analysis. São Paulo City, São Paulo State, Brazil, 2020.

Item		Delphi 1		Delphi 2		Delphi 3	
		Agreement (%)	CVI	Agreement (%)	CVI	Agreement (%)	CVI
I-1	Relevance	100	1	100	1	100	1
	Clarity	90	0.97	88	0.95	100	1
I-2	Relevance	100	1	100	1	100	1
	Clarity	90	0.97	88	0.95	100	1
I-3	Relevance	80	0.93	75	0.91	88	0.95
	Clarity	40	0.77	38	0.75	88	0.95
I-4	Relevance	100	1	100	1	100	1
	Clarity	90	0.97	88	0.95	100	1
	Final classification	100	1	100	1	100	1
	Score of items	90	0.97	100	1	88	0.95

Source: elaborated by the authors. CVI: content validity index.

In the first evaluation cycle, most items showed acceptable agreement values and CVI as to relevance and clarity. However, in item three, only 40% of the judges agreed on its clarity. Initially, item three was divided into three groups, with scores from zero to two, each group containing a list of diseases and/or clinical condition according to severity. Three judges suggested revising the list, two proposed changing disease scores, and two, dividing clinical and surgical diseases. Thus, the item was reformulated according to the suggestions and submitted to a second evaluation cycle.

 In this second cycle, item three of the tool, referring to neonate’s disease and/or clinical condition, again there was no consensus, showing low agreement between the judges as to clarity. Experts stated that the item could generate doubts in the classification, and some suggested non-applicable changes due to the lack of practicality, such as attaching a list of diseases and/or clinical conditions to the tool. In view of the results and suggestions, some pertinent changes were made, and there was a dialogue with the four judges separately to clarify doubts about the tool’s objective, which was then sent to the third evaluation cycle. After this cycle, calculations of agreement rate and CVI were performed, reaching the consensus values initially proposed. Thus, the final version of the tool was made available.

With the tool validated, four evaluators were invited to apply it to a group of 16 newborns and classify them as being of low, medium, or high nutritional risk, like it is shown in Table 3. In this stage of the study, the tool’s internal consistency was verified with Cronbach’s *alpha*, obtaining a value of 0.96, which translates as almost perfect consistency. In order to verify agreement among professionals, Fleiss’ kappa was used, obtaining k=0.74, which means good agreement among evaluators, with a value of p <0.001.

**Table 3:** Application of the Neonatal Nutritional Risk Screening Tool in 16 newborns admitted to Neonatal Intensive Care Center 2 (NICC2). São Paulo City, São Paulo State, Brazil, 2020.

Patient	Evaluator 1	Evaluator 2	Evaluator 3	Evaluator 4	% HNR	% MNR	% LNR
1	LNR	MNR	MNR	MNR	0	75	25
2	HNR	HNR	HNR	HNR	100	0	0
3	HNR	HNR	HNR	HNR	100	0	0
4	HNR	HNR	HNR	HNR	100	0	0
5	MNR	MNR	MNR	MNR	0	100	0
6	HNR	HNR	HNR	HNR	100	0	0
7	LNR	MNR	MNR	MNR	0	75	25
8	HNR	HNR	HNR	HNR	100	0	0
9	HNR	HNR	HNR	HNR	100	0	0
10	LNR	LNR	LNR	LNR	0	0	100
11	MNR	MNR	MNR	MNR	0	100	0
12	MNR	MNR	HNR	MNR	25	75	0
13	HNR	HNR	HNR	HNR	100	0	0
14	HNR	HNR	HNR	HNR	100	0	0
15	HNR	MNR	MNR	MNR	25	75	0
16	LNR	LNR	MNR	LNR	0	25	75

Source: elaborated by the authors. LNR: low nutritional risk; MNR: medium nutritional risk; HNR: high nutritional risk.

In the application of the aforementioned tool, eight of the 16 patients were classified as high nutritional risk, two as medium nutritional risk, one as low nutritional risk, and five obtained two nutritional risk ratings by the evaluators, as shown in Table 3. The ratings reflect, mainly, the characteristics of the services in which the tool was applied, considering that they preferentially assist patients with complex pathologies, such as diaphragmatic hernia, gastroschisis, among others.

**Chart 1:**
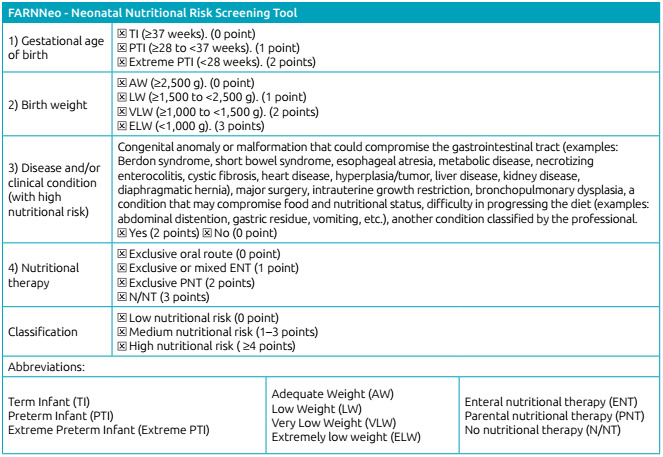
Neonatal Nutritional Risk Screening Tool.

After validating both the tool’s content applicability, FARNNeo was obtained, presented in Chart 1.

## DISCUSSION

The present study built and validated the nutritional risk screening tool, FARNNeo, for use in neonates admitted to NICC. The nutritional risk screening tools are extremely important in the hospital environment and should be used at the time of admission, because they can early identify factors that affect nutritional status.^9^ ^,^ ^19^ ^,^ ^20^

The tools for nutritional screening of patients admitted to the NICC are scarce and, in many situations, those that exist are not suitable for Brazilian NICCs. An example is the Neonatal Nutrition Screening Tool (NNST), which uses a test that checks diastolic flow of the umbilical artery as one of its criteria, a parameter that is not routinely adopted in Brazilian public hospitals.^21^ Another existing tool for neonates admitted to NICC is the Ohio Neonatal Nutritionists’ Screening, which establishes criteria to identify newborns with high nutritional risk using biochemical tests as one of its parameters, making the tool impractical.^22^ There is also the Clinical Assessment of Nutrition Score (CANSCORE), which assesses the newborn’s nutritional status using anthropometric measurements, including their arm circumference. This parameter is not routinely adopted in the NICC, which makes the tool impractical, in addition to having the objective of identifying malnutrition and predicting associated neonatal morbidity, instead of establishing nutritional support.^23^ For a screening tool of nutritional risk to be considered adequate, it must: measure what it proposes to assess, be practical, simple, effective, and amenable to application by a health professional, be it a nutritionist, nurse or doctor.^19^ ^,^ ^24^

During the validation process, eight judges participated in the critical analysis of the tool. This is the number recommended in literature, which suggests a minimum of five and a maximum of 10 judges to participate in the validation process.^25^

After content validation, a group of professionals applied FARNNeo to verify its reproducibility and internal consistency. Several studies use these parameters to measure agreement among evaluators, using Kappa and the reliability of internal consistency with Cronbach’s *alpha* coefficient. Results interpretation has no consensus, and researchers can define their parameters, but the ideal is that the values are not under 0.7.^16^ ^,^ ^25^

When applying FARNNeo, each service can define the systematization of care according to risk classification, better allocating resources and offering an adequate and great nutritional support.^26^

Some NICC have limited human resources and a high number of NBs to be assisted, which makes effective nutritional care impossible. Thus, screening can assist in the monitoring of newborns and guide the needed nutritional management, so that the group at highest risk is evaluated with greater attention and frequency by the specialized clinical team.^26^ ^,^ ^27^

The present study has some limitations, such as the fact that it was unable to include judges from different regions of the country, restricting itself to local knowledge and practices. Besides that, most judges work in the hospital where the study was conducted, apart from one. Another limitation is that, although the tool is adequate in terms of relevance and clarity of the items for nutritional screening of newborns and it has good reproducibility, further tests that relate the nutritional risk found in the tool and the development of newborns during hospitalization and its outcomes are needed. This would allow researchers to analyze if the classification of the risk found matches the clinical and nutritional conditions of the newborn.

Studying the application of FARNNeo in more patients is encouraged to verify the relation between the classification of nutritional risk, its behaviors, and outcomes.

The tool created and validated proved to be easy to apply, and presented, with the evaluations of judges, reliability. Thus, it provides subsidies to be used regularly by professionals in hospitals, such as screening for early nutritional risk and systematizing care, so that more qualified care is offered.
